# Handing the Microphone to Women: Changes in Gender Representation in Editorial Contributions Across Medical and Health Journals 2008-2018

**DOI:** 10.15171/ijhpm.2020.06

**Published:** 2020-01-20

**Authors:** Angela Y. Chang, Nina Cesare

**Affiliations:** ^1^Institute for Health Metrics and Evaluation, University of Washington, Seattle, WA, USA.; ^2^Boston University School of Public Health, Boston, MA, USA.

**Keywords:** Gender Equality, Publication, Bias, Editorials

## Abstract

The editorial materials in top medical and public health journals are opportunities for experts to offer thoughts that might influence the trajectory of the field. To date, while some studies have examined gender bias in the publication of editorial materials in medical journals, none have studied public health journals. In this perspective, we studied the gender ratio of the editorial materials published in the top health and medical sciences journals between 2008 and early 2018 to test whether gender bias exists. We studied a total of 59 top journals in health and medical sciences. Overall, while there is a trend of increasing proportion of female first authors, there is still a greater proportion of male than female first authors. The average male-to-female first author ratio during the study period across all journals was 2.08. Ensuring equal access and exposure through journal editorials is a critical step, albeit only one step of a longer journey, towards gender balance in health and medical sciences research. Editors of top journals have a key role to play in pushing the fields towards more balanced gender equality, and we strongly urge editors to rethink the strategies for inviting authors for editorial materials.

Similar to opinion pieces in the New York Times, the editorial materials in top medical and public health journals are opportunities for experts to offer thoughts that might influence the trajectory of the field. Authors of the editorials are commonly those who are considered as authoritative figures in their domains or key stakeholders, such as officials from national governments or international organizations. For some journals, editorial pieces are commissioned and elicited through invitations from editors, while others are open to everyone.

We believe who the editors hand the microphone to matters, as they have the power to shift the focus of future research. Given this influence, it is critical that the authors who contribute this work are not only well-informed but able to contribute a unique viewpoint to current work. Representation of a wide range of perspectives in editorials are important for a constructive conversation. Researchers from different backgrounds and identities based on factors such as gender, geography, profession, race, and culture carry unique perspectives that should be heard in current literature and can shape future work for the better.^1,2^

Publications in response to a 2014 Ebola outbreak illustrated the importance of incorporating diverse professional perspectives in editorial content. Most editorials published at that time focused on the general emergency response in west Africa. However, experts in women’s health called for more attention to women’s reproductive health and maternal mortality.^3,4^ A humanitarian worker raised the importance of handling survivors with respect and care, highlighting possible community stigma for those diagnosed as pathogen carriers.^5^ Anthropologists used the framework of the prisoner’s dilemma to illustrate how, given the infectious nature of Ebola and sanitation issues associated with Ebola clinics, those who are suspected of being Ebola positive experience high mortality rates for seemingly positive outcomes (eg, visiting a clinic and finding out they are not Ebola positive but contracting the disease nonetheless, or electing to stay home and, despite being infected thus missing an opportunity to treat the disease).^6^

While promoting diversity in science may be seen as the ‘moral and ethical duty’ of the medical research community, there is nonetheless evidence that demographic imbalances in the production and distribution of research exist. Existing studies on gender balance in scientific research have identified evidence of gender bias in professional hiring, mentoring, and promotion.^7^ Regarding publication, women tend to be in a more disadvantageous state than men.^8^ Filardo et al studied the patterns and trends of female first authors among six top medical journals and found overall lower proportions of female first authors than men in 2014, though this ratio has improved over time.^9^ Others have found that papers with women as first- or solo-author positions received fewer citations than men with the same positions.^10^

A journal editorial, broadly speaking, is a concise, scholarly statement that discusses issues, policies or scientific discoveries of interest to the readership. The editorial analyzes evidence rather than produces it, and is intended to shape opinions of those in the field.^11^ Nittrouer et al define the concept of gender gatekeepers as individuals who make decisions about who will advance through their careers, and may, often unintentionally, create gender disparities.^12^ In this perspective, we view journal editors as the “gatekeepers” that decide who gets to voice their opinions on their platforms. There is a strong potential for gender bias in editorials, as studies have shown that people (of both genders) tend to view men with higher expertise than women. For example, both men and women rate identical scientific abstracts or applications for a laboratory manager’s position as of higher quality when these are submitted under a male rather than female name, link science words more quickly with male than female names, and give harsher reviews to female authored submissions.^13-16^ Another study found that after introducing a double-blind review policy, one journal saw a significant increase in female first-authored papers.^17^

To date, while some have examined gender bias in the publication of editorial materials in both medical journals, no one has systematically studied biases in public health journals.^18-20^ To fill this gap, in this perspective, we studied the gender ratio of the editorial materials published in the top health and medical sciences journals between 2008 and early 2018 to test whether gender bias exists. Compared to existing studies, focusing solely on editorial materials allows us to remove potential confounders, such as paper quality, type of study (clinical trials, observational studies), and funding sources. We assume that the authors in our studies were selected from a pool of highly qualified experts, and therefore minimize the likelihood that the gender bias originates from elsewhere outside of the journals themselves.

We selected a total of 59 top journals in health and medical sciences, and downloaded the metadata on all editorial materials published between 2008 to April 2018 from Thomson Reuters’ Web of Science database, listed in Supplementary file 1.We collected the list of all editors from the journals’ respective websites. We extracted the names of editors in chief and all editors who likely have decisive functions regarding manuscript acceptance. Editorial materials with any of the journal editors listed as the first author were excluded. Given that in medical literature the first author is the author who contributes most significantly,^21^ we extracted the first names of the first authors from all papers included in the dataset, and estimated authors’ gender using the gender package in R, which leverages historical data from sources such as the US Census or Social Security Administration to estimate whether first names are likely male or female.^22^ To quantify uncertainty in our automated gender classification, we selected a random sample of 100 names from the articles, assigned gender for each based on Google search results, and assessed the performance of the gender classifier on this sample. We found 86% to be accurately classified, and the remaining unclassified. This suggests that, when a gender is assigned to a name by the classifier, we can be highly confident about the result.

Across all journals, 57 197 editorial materials met our criteria and were included in the analysis. We were able to assign genders to the first names of first authors of 86.3% of papers using the R package. Among the remaining 13.7%, we randomly selected 10% and determined the gender via manual search.

Overall, there is a greater proportion of male than female first authors across majority of journals. Public health journals, on average, are estimated to have higher proportion of female first authors than journals in other fields, although the estimated average ratio is still above one, suggesting that more male authors are publishing editorials. The average male-to-female first author ratio between 2008 to April 2018 across all journals was 2.08. *MMWR-Morbidity and Mortality Weekly Report* had the lowest gender ratio, at 0.4 (ie, there were 2.5 times more female first authors in the editorial materials than males), followed by *Journal of Adolescent Health* (0.8), *Environmental Health Perspectives* (0.9), *Health Affairs* (1.0), and *American Journal of Public Health* (1.1) (Figure 1). On the other end of the spectrum, we estimate that *Deutsches Arzteblatt International* had the highest ratio of 9.0, followed by *Nature Genetics* (3.5), *The Lancet Oncology* (3.5), *Annals of Internal Medicine* (3.0), and *Blood* (2.9). US-based journals have an average ratio of 1.91, while UK-based journals have a slightly higher average of 2.04 (figure by country is in Supplementary file 1). Life sciences journals have the highest average gender ratio (2.39), followed by medical (2.27), healthcare/other (1.69), and public health journals (1.65) (Supplementary file 1).

**Figure 1:**
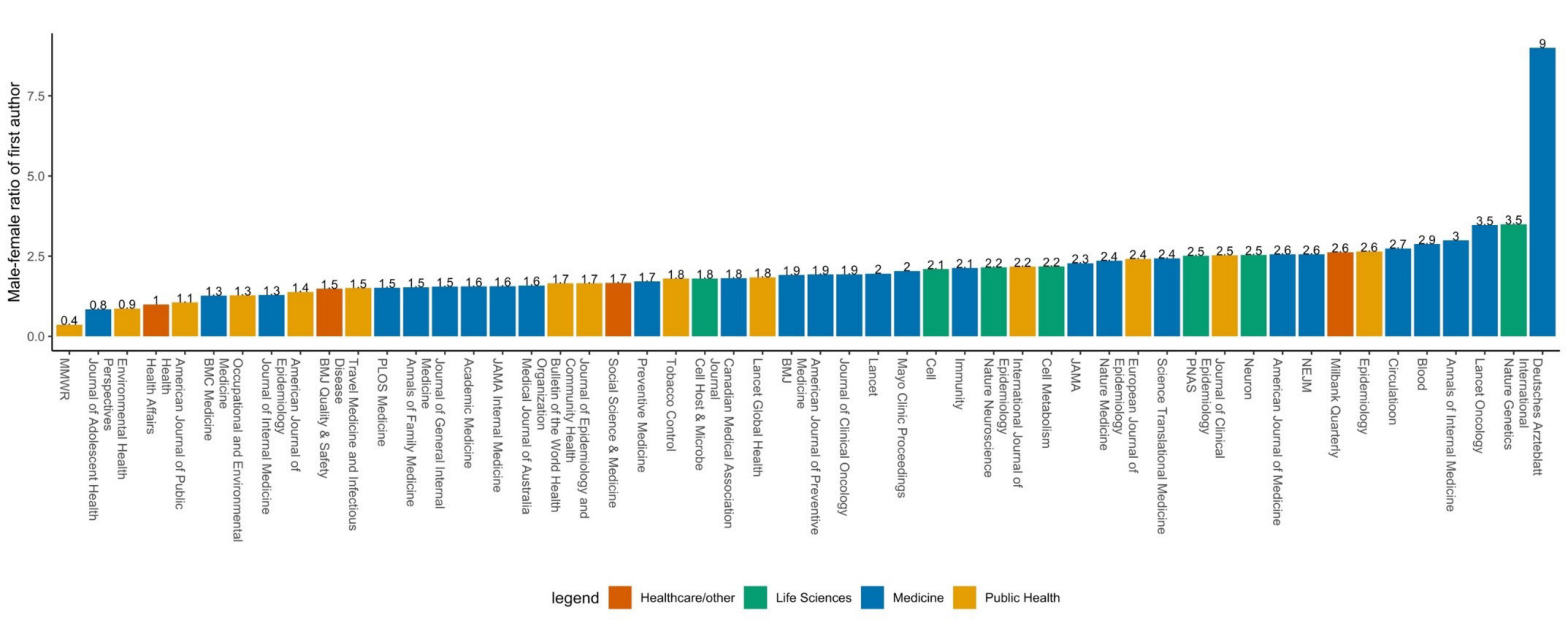
Average Ratio of Male-to-Female First Authors Between 2008 and April 2018, by Field.

Focusing on the more recent full year, in 2017, we estimate that most journals have lower male-to-female first author ratios than the average across the study period, with an average of 1.78 across all journals (Figure 2). Journals with the lowest ratios are: *MMWR-Morbidity and Mortality Weekly Report* (0.3), *Health Affairs* (0.4), *Preventive Medicine* (0.6), *Journal of Adolescent Health* (0.6), and *Environmental Health Perspectives* (0.8). Journals with the highest ratios include *Deutsches Arzteblatt International* (7.8), *Science Translational Medicine* (5.7), *Milbank Quarterly* (2.8), *Circulation* (2.8), and *Blood* (2.8). UK-based journals have slightly lower estimated gender ratios (1.64) than US-based journals (1.70), and medical journals (1.98) have the highest ratio in 2017, compared to life sciences (1.88), healthcare/other (1.70), and public health journals (1.36) (Supplementary file 1). Finally, comparing across time, we estimate decreased ratio in 2017 compared to the average of the ten years, in 38 journals, while 14 of the following journals had higher ratio in 2017 than the full study period.

**Figure 2:**
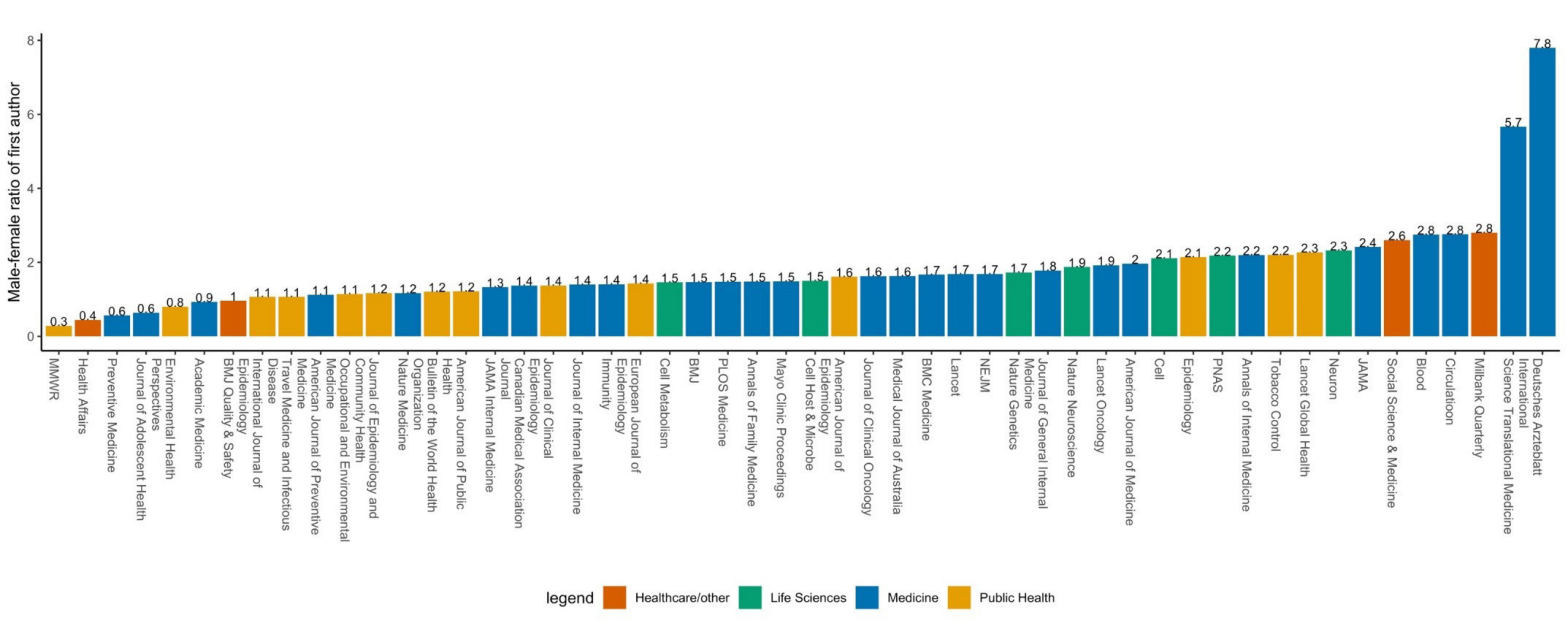
Ratio of Male-To-Female First Authors in 2017 (the Most Recent Full Year), by Field.

The gender imbalance identified in this commentary may reflect the fact that the pool of experts and researchers in health and medical sciences consists mostly of men. For example, in both academia and politics, the ratio of women to men tend to decrease as we climb up the career ladder.^23^ To explore this, we accessed data from the American Association of Medical Colleges on overall faculty gender ratios and faculty gender ratios by rank. Using the proportion of female faculty members for a select year (2017) as our probability of an editor selecting an author without regard to gender, we generate 1000 possible contributor pools of 100 authors each. The average male-to-female author ratio if we consider faculty at all ranks is 1.51 [CI: 1.49-1.53]. If we consider only senior faculty members, it is 3.39 [95% CI: 3.33, 3.44]. The average male-female gender ratio of US Journals in our data is 1.91, which suggests that US authored commentaries are more frequently authored by males than we would expect in absence of gender bias. While this ratio may be shaped by gender bias, it may also reflect a tendency to select more senior faculty members – a large proportion of whom are male (76.1% as of 2017).

Beyond the possibility of skewing invitations toward senior faculty members, if journal editors send out equal numbers of invitations to men and women, the former may tend to accept the invitations more frequently than women, leading to more editorials written by men. Similarly, men may be more willing to voice their opinions by submitting more than women. This study cannot disentangle whether journals are simply reflecting the gender bias within the field of health and medical sciences or are in part exacerbating gender imbalance.

Establishing a gender balance in publishing may create effects that promote gender equality in the sciences overall. Publishing an editorial in a high-impact journal itself is a signal of authority. At the population level, seeing more men publish editorials may convey to readers that men have more authority in this field than women, further augmenting the gender bias. Furthermore, the role model effect, which suggests that the presence of female leadership is positively associated with better performance in junior populations, have been widely documented.^24,25^ At the individual level, having an editorial in a reputable journal such as the Lancet lends credibility to the author, which may lead to more funding or engagement opportunities,^26^ an area in which women typically lag behind men,^23,27-29^ and even when rewarded, women tend to receive less in terms of award value. Similarly, when organizing conferences or workshops, organizers may also invite names seen in top journals, further advancing their reputation. Overall, inviting more women to contribute these high-profile pieces may lead to more female representation across contexts.

Based on these findings, we believe that more diverse gender representation is needed in health research. Our call for more gender balance in editorial materials is in line with projects advocating for gender equity in science (such as European Commission’s Horizon 2020 and the special issue on the Lancet on advancing women in science, medicine, and global health^30,31^). In academic health research, the lack of gender and racial diversity in leadership positions in academic research have been highlighted as an urgent matter by several prominent organizations, including the Institute of Medicine, European Commission, National Institute for Health, and the American Association of Medical Colleges.^30,32-34^ Increasing female representation in science will require institutional changes, such as providing mentorship to early career female researchers and addressing the ways in which systemic impacts opportunities for career advancement.

Ensuring equal access and exposure through journal editorials is a critical step, albeit only one step of a longer journey, towards gender balance in health and medical sciences research. Editors of top journals have a key role to play in pushing the fields towards more balanced gender equality, and we strongly urge editors to rethink the strategies for inviting authors for editorial materials.

## Ethical issues

Not applicable.

## Competing interests

Authors declare that they have no competing interests.

## Authors’ contributions

Both authors were involved in the conception, analses, drafting, and revision of the paper.

## Authors’ affiliations

^1^Institute for Health Metrics and Evaluation, University of Washington, Seattle, WA, USA. ^2^Boston University School of Public Health, Boston, MA, USA.

## Supplementary files


Supplementary file 1 contains Table S1, Figure S1, and Figure S2.
