# Supplementary material for: Handing the Microphone to Women: Changes in Gender Representation in Editorial Contributions Across Medical and Health Journals 2008-2018
Source: Int J Health Policy Manag. 2020 Jan 20;9(7):269–73. doi: 10.15171/ijhpm.2020.06 (PMC7444434; doi:10.15171/ijhpm.2020.06)
Supplement: Supplementary file 1 — contains Table S1, Figure S1, and Figure S2. [file ijhpm-9-269-s001.pdf]

Supplementary file 1

Methods

We considered two sets of top journals in health and medical sciences. The first set comes from Google Scholar's two sets of rankings of top 20 health and medical sciences journals in April 2018, of which 32 unique journal names were present and considered for the analysis (Table S1). The second set comes from four sets of rankings of the Web of Science's InCites Journal Report Citation website, including 1) medicine (general and internal), 2) public, environmental, and occupational health, 3) healthcare science and services, and 4) health policy and services. These four sets of rankings included 47 unique journal names, all of which were included in the analysis.

For all 79 journals, we downloaded the metadata on all editorial materials published between 2008 to April 2018 from Thomson Reuters' Web of Science database. Journals not listed in Web of Science, as well as journals with less than 100 editorial materials during the study period were excluded, the latter due to small numbers of editorials published each year, making the estimation of the ratios unreliable. Editorial materials are listed under different sections in each journal, such as "Comment" in the Lancet, "Commentary" in the New England Journal of Medicine, and "Opinion" and "Viewpoint" in JAMA. To ensure consistency, we searched for articles classified under "editorial materials" under Web of Science, which editorial materials to include editorials, interviews, commentary, and discussions between individual, post-paper discussions, round table symposia, and clinical conferences,[35] but not letters or correspondences from the readers to the journal editor concerning previously published articles. To ensure that the metadata includes primarily editorial materials and not clinical case reports (papers that describe clinical cases that may be of interest to the clinical community),[36] we first downloaded the metadata of case reports from the Web of Science and removed listed articles from our data. 52 of the 79 journals matched our inclusion criteria and were included in this analysis, listed in Table S1.

For each journal, we first assigned a country of origin based on information provided on the journals' or publishers' websites. For many, the websites did not specify the country of origin, so we used the country in which the publisher is based instead (see Table S1). We determined whether

the journal is a medical, public health, life sciences, or healthcare/other journal based on the journal's own description or Wikipedia, which specifies whether the journal is a "medical," "scientific," "public health," or "healthcare" journal. We then collected the list of all editors from the journals' respective websites. We extracted the names of editors in chief and all editors who likely have decisive functions regarding manuscript acceptance. This includes senior (executive) editors, acting chief editor, executive editors, deputy editors, associate editors, regional editors, research editors, (senior) managing editors, scientific editors, and section-specific editors (such as healthcare policy and law editors, news editors, perspective editors, letters editors, editorials editors). We excluded editorial staff members, such as editorial assistants, technical editors, images editors, digital content editors, assistant web editors, multimedia editors, and statistical editors, as well as honorary editors such as editors emeritus, honorary deputy editor, senior guest editor, and founding editors. Editorial materials with any of the journal editors listed as the first author were excluded (see list of journal editors in Supplementary file 1).

We extracted the first names of the first authors from all papers included in the dataset. We estimated authors' gender using the gender package in R, which relies on U.S. Census or Social Security datasets. To quantify uncertainty in our automated gender classification, we selected a random sample of 100 names from the articles, assigned gender for each based on Google search results, and assessed the performance of the gender classifier on this sample. We found 86% to be accurately classified, and 14% to remain as unclassified. This suggests that, when a gender is assigned to a name by the classifier, we can be highly confident about the result. Among papers with names that were not assigned a gender, we randomly selected 10% of papers and manually searched for the gender of the author based on the full name and the institution to which the author belongs to through Google.

Limitations

First, this study is descriptive in nature, and we cannot determine the factors that lead to the findings of higher male-to-female first authors. Second, we were unable to find the exact processes in which journal editors determine the content of the editorials: who decides the topics of the editorials, whom to invite, and which editorials to publish. The instructions listed on most journals' websites state that most editorials are invitation-only or not, but do not discuss which route is more

common. Third, we only looked at the years 2008-2018 and not earlier years because the metadata from Web of Science did not include full first-names in prior years. We were only able to obtain the names of journal editors in April 2018 and not prior years, therefore limiting our study to recent years reduces the possibility that previous editors authored some of the editorials. Fifth, we only analyzed the gender of the first author, who we assumed is the main author of the article, instead of the senior author or all co-authors.

Table S1. Summary Statistics

Source	Journal [1]	Research field [2]	Number of editorial materials included in analysis [3]	Editor-in -chief/editor	Journal's country of origin	Estimated percentage of gender of first author unknown	Estimated ratio of male-to-female first authors
Health care Sciences & Service (WoS)	Academic Medicine	Medicine	1,163	David P. Sklar	US	9.2	1.56
Public, Environmental & Occupational Health (WoS)	American Journal of Epidemiology	Public Health	412	Moyses Szklo	US	13.8	1.38
Medicine -- General & Internal (WoS)	American Journal of Medicine	Medicine	1,063	Joseph Alpert	US	18.2	2.56
Medicine -- General & Internal (WoS)	American Journal of Preventive Medicine	Medicine	378	Matthew L. Boulton	US	7.1	1.93
Public, Environmental & Occupational Health (WoS)	American Journal of Public Health	Public Health	1,294	Alfredo Morabia	US	9.0	1.06
Medicine -- General & Internal (WoS)	Annals of Family Medicine	Medicine	466	Kurt C. Stange	US	7.1	1.53
Health & Medicine (Google Scholar)	Annals of Internal Medicine	Medicine	2,420	Christine Laine	US	14.4	2.99
Health & Medicine (Google Scholar)	Blood	Medicine	2,721	Bob Lowenberg	US	16.2	2.88
Health & Medicine (Google Scholar)	BMC Medicine	Medicine	343	Lin Lee	UK	18.3	2.87

Health care Sciences & Service (WoS)	BMJ Quality & Safety	Healthcare /other	267	Kaveh G Shojania<br>Mary Dixon-Woods	UK	8.6	1.49
Health & Medicine (Google Scholar)	British Medical Journal	Medicine	4,555	Fiona Godlee	UK	14.7	1.88
Public, Environmental & Occupational Health (WoS)	Bulletin of the World Health Organization	Public Health	535	Laragh Gollogly	Switzerland	22.0	1.65
Medicine -- General & Internal (WoS)	Canadian Medical Association Journal	Medicine	1,829	Diane Kelsall	Canada	14.4	1.81
Health & Medicine (Google Scholar)	Cell	Life sciences	1,502	Emilie Marcus [4]	US	16.1	2.10
Health & Medicine (Google Scholar)	Cell Host & Microbe	Life sciences	523	Lakshmi Goyal	US	17.2	1.80
Health & Medicine (Google Scholar)	Cell Metabolism	Life sciences	550	Nikla Emambokus	US	15.1	2.18
Health & Medicine (Google Scholar)	Circulation	Medicine	2,698	Joseph A. Hill	US	15.3	3.47
Medicine -- General & Internal (WoS)	Deutsches Arzteblatt International	Medicine	180	Egbert Maibach-Nagel	Germany	13.3	9.00
Public, Environmental & Occupational Health (WoS)	Environmental Health Perspectives	Public Health	640	Sally Perreault Darney	US	5.8	0.86
Public, Environmental & Occupational Health (WoS)	Epidemiology	Public Health	299	Timothy L. Lash	US	12.7	2.65
Public, Environmental & Occupational Health (WoS)	European Journal of Epidemiology	Public Health	140	Albert Hofman	Germany [5]	25.7	2.41

Health care Sciences & Service (WoS)	Health Affairs	Healthcare /other	404	Alan Weil	US	6.9	0.99
Health & Medicine (Google Scholar)	Immunity	Medicine	650	Peter Lee	US	18.9	2.13
Public, Environmental & Occupational Health (WoS)	International Journal of Epidemiology	Public Health	822	Stephen Leeder	UK [5]	17.8	2.17
Health & Medicine (Google Scholar)	JAMA – Journal of the American Medical Association	Medicine	3,758	Howard Bauchner	US	7.8	2.45
Health & Medicine (Google Scholar)	JAMA Internal Medicine	Medicine	961	Rita F. Redberg	US	7.8	1.56
Public, Environmental & Occupational Health (WoS)	Journal of Adolescent Health	Medicine	326	Charles E. Irwin, Jr.	US [5]	11.4	0.84
Public, Environmental & Occupational Health (WoS)	Journal of Clinical Epidemiology	Public Health	233	Andre Knottnerus, Peter Tugwell	US [5]	20.2	2.53
Health & Medicine (Google Scholar)	Journal of Clinical Oncology	Medicine	1,633	Stephen A. Cannistra	US	11.3	1.93
Public, Environmental & Occupational Health (WoS)	Journal of Epidemiology and Community Health	Public Health	254	Martin Bobak James R. Dunn	UK	26.8	1.65
Medicine -- General & Internal (WoS)	Journal of General Internal Medicine	Medicine	1,211	Steven M Asch, Carol Bates, Jeffrey L Jackson	US	11.1	1.55
Medicine -- General & Internal (WoS)	Journal of Internal Medicine	Medicine	174	Ulf de Faire	US [5]	91.4	1.29

Public, Environmental & Occupational Health (WoS)	Lancet Global Health, The	Public Health	522	Zoe Mullan	UK	18.8	1.84
Health & Medicine (Google Scholar)	Lancet Oncology, The	Medicine	1,538	David Collingridge	UK	15.0	3.48
Health & Medicine (Google Scholar)	Lancet, The	Medicine	5,381	Richard Horton	UK	13.8	1.95
Medicine -- General & Internal (WoS)	Mayo Clinic Proceedings	Medicine	768	Karl A. Nath	US	11.7	2.04
Medicine -- General & Internal (WoS)	Medical Journal of Australia	Medicine	2,155	Nick Talley	Australia	7.9	1.59
Health care Sciences & Service (WoS)	Milbank Quarterly	Healthcare /other	181	Alan B. Cohen	US	-	2.62
Public, Environmental & Occupational Health (WoS)	MMWR-Morbidity and Mortality Weekly Report	Public Health	347	Charlotte K. Kent	US	19.0	0.36
Health & Medicine (Google Scholar)	Nature Genetics	Life sciences	373	Myles Axton	UK	17.7	3.49
Health & Medicine (Google Scholar)	Nature Medicine	Medicine	916	Joao Monteiro	UK	13.8	2.36
Health & Medicine (Google Scholar)	Nature Neuroscience	Life sciences	537	Kevin Da Silva	UK	16.8	2.16
Health & Medicine (Google Scholar)	Neuron	Life sciences	1,062	Mariela Zirlinger	US	17.1	2.54
Health & Medicine (Google Scholar)/	New England Journal of Medicine, The	Medicine	4,380	Jeffrey M. Drazen	US	12.0	2.56

Public, Environmental & Occupational Health (WoS)	Occupational and Environmental Medicine	Public Health	171	Malcolm Sim	UK [5]	15.2	1.28
Health & Medicine (Google Scholar)	PLOS Medicine	Medicine	762	Larry Peiperl	US	15.0	1.51
Medicine -- General & Internal (WoS)	Preventive Medicine	Medicine	198	Eduardo L. Franco	US [5]	12.1	1.71
Health & Medicine (Google Scholar)	Proceedings of the National Academy of Sciences	Life sciences	2,507	Natasha V. Raikhel	US	15.8	2.51
Health & Medicine (Google Scholar)	Science Translational Medicine	Medicine	440	Jeremy Berg	US	18.4	2.43
Health & Medicine (Google Scholar)	Social Science & Medicine	Healthcare /other	232	Ichiro Kawachi<br>S.V. Subramanian	US [5]	12.5	1.67
Public, Environmental & Occupational Health (WoS)	Tobacco Control	Public Health	154	Ruth Malone	UK [5]	21.4	1.80
Public, Environmental & Occupational Health (WoS)	Travel Medicine and Infectious Disease	Public Health	169	Patricia Schlagenhauf-Lawlor	US [5]	20.1	1.51

1. The following journals were excluded from the analysis due small numbers of editorial materials (less than 100) during the study period: AIDS Patient Care and STDs, Amyloid – Journal of Protein Folding Disorders, Annual Review of Public Health, Cancer Epidemiology Biomarkers & Prevention, The Cochrane Database of Systematic Reviews, Cold Spring Harbor Perspectives in Medicine, Epidemiologic Reviews, Environmental Research, The FASEB Journal, Implementation Science, Indoor Air, International Journal of Hygiene and Environmental Health, International Journal of Molecular Sciences, JMIR mHealth and

uHealth, Journal of Cachexia Sarcopenia and Muscle, Journal of Clinical Medicine, Journal of Global Health, Journal of Medical Internet Research, Journal of Toxicology and Environmental Health – Part B – Critical Reviews, Nicotine & Tobacco Research, Translational Research, Palliative Medicine, PLOS One, Value in Health.

The following journals were excluded from the analysis due to lack of data availability in Web of Science: Journal of the American College of Cardiology, The Journal of Clinical Investigation.

2. Research field was determined based on the journals' own descriptions or Wikipedia.
3. Excluding editorials with any of the editors as the first author and case reports.
4. Emilie Marcus was editor in chief until December 2017.
5. The journal's country of origin was not listed on its website. We instead assigned the publisher's country of origin.

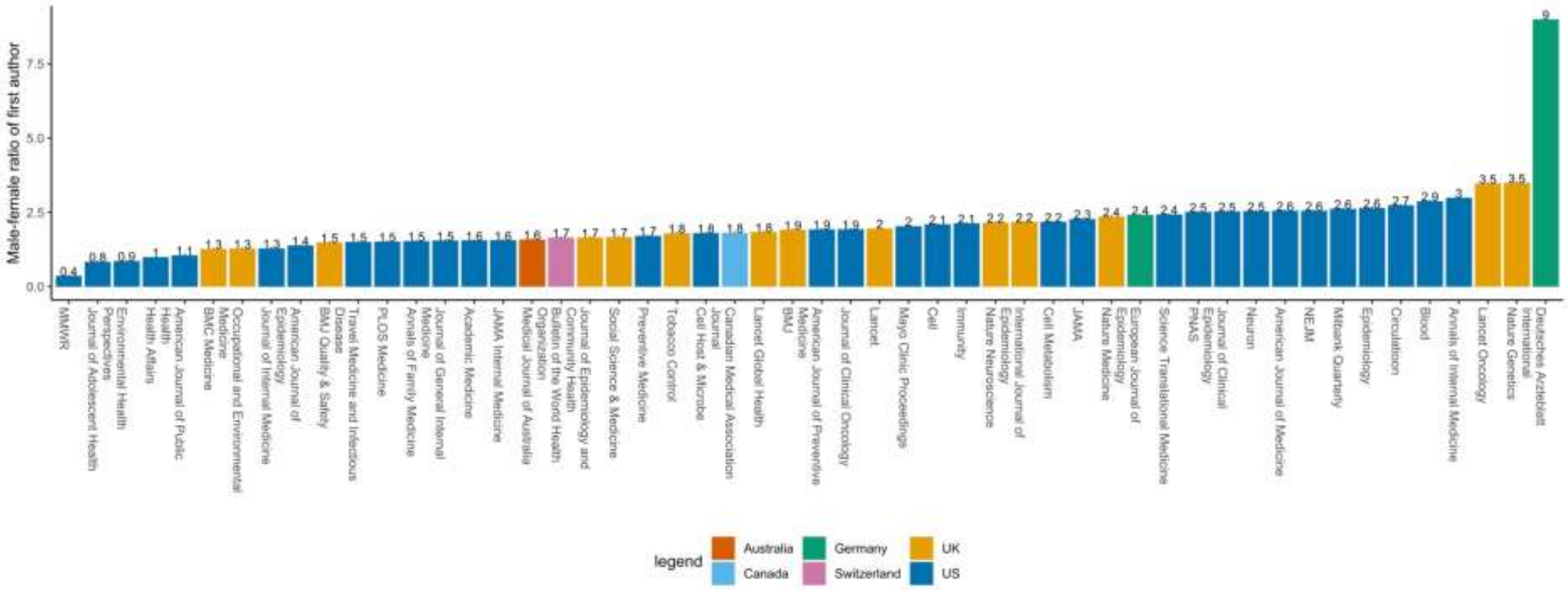

Figure S1. Average Ratio of Male-to-Female First Authors Between 2008 and April 2018, by Country.

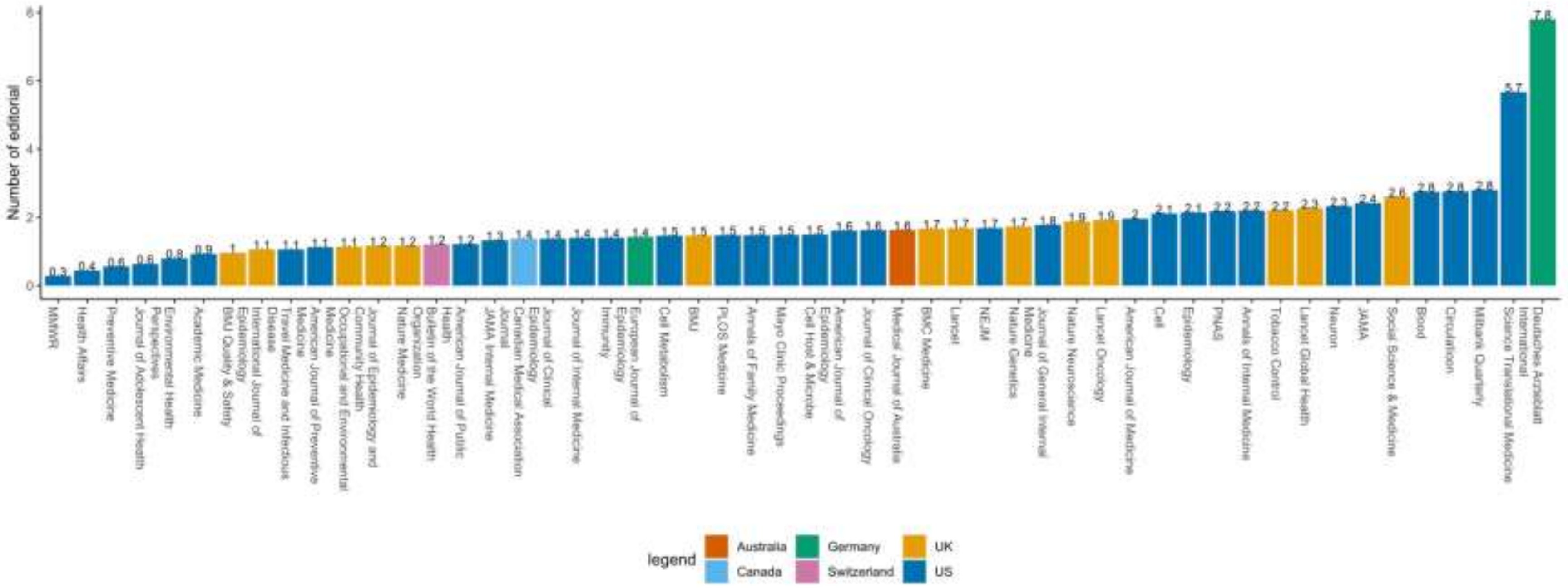

Figure S2. Ratio of Male-to-Female First Authors in 2017 (the Most Recent Full Year), by Country.
